# Inhibition of SIK1 Alleviates the Pathologies of Psoriasis by Disrupting IL-17 Signaling

**DOI:** 10.1155/mi/3540219

**Published:** 2025-02-07

**Authors:** Dongxuan Huang, Huimin Sun, Lianhui Su, Fan Yang, Dongsheng Huang, Hanchao Gao, Mengtao Cao

**Affiliations:** ^1^Department of Respiratory Medicine, Shenzhen Longhua District Central Hospital, Shenzhen 518110, China; ^2^Department of Medical Laboratory, Shenzhen Longhua District Central Hospital, Shenzhen, Guangdong 518110, China; ^3^Department of Nephrology, Shenzhen Longhua District Central Hospital, Shenzhen Longhua District Key Laboratory for Diagnosis and Treatment of Chronic Kidney Disease, Shenzhen 518110, China; ^4^Department of Clinical Laboratory, Shenzhen Second People's Hospital, First Affiliated Hospital of Shenzhen University, Shenzhen, Guangdong 518110, China

## Abstract

Psoriasis is an inflammatory skin disease mediated by multiple immune cells, including T cells, macrophages, and dendritic cells, which exhibit complex pathologies and limited clinical treatment. Here, we found that salt-inducible kinase 1 (SIK1) was upregulated in the imiquimod (IMQ)-induced psoriasis mouse model. This increment may be due to a higher level of interleukin-17, which promoted the expression of SIK1 in keratinocytes. Inhibition of SIK1 kinase activity using a small molecular inhibitor (HG-9-91-01 or YKL-06-062) dramatically alleviated IMQ-induced psoriasis, showing reduced epidermal thickness, inflammation, and hyperproliferative epidermal keratinocytes. Our data demonstrated that SIK1 inhibitors HG-9-91-01 or YKL-06-062 blocked the expression of IL-17-induced proinflammatory cytokines and chemokines, including *Il6*, *Kc*, and *Ccl20*. Mechanistically, we found that SIK1 inhibitor HG-9-91-01 or YKL-06-062 suppressed the phosphorylation of I*κ*b*α* and P38. Consistently, SIK1 overexpression in keratinocytes promoted the activation of I*κ*b*α* and P38. Collectively, our results reveal that SIK1 participates to promote IL17-induced signaling through enhancing activation of NF-*κ*B and MAPKs and exacerbates psoriasis-like skin inflammation. Thus, inhibition of SIK1 presents a potential new therapeutic target for psoriasis.

## 1. Introduction

Psoriasis is a chronic inflammatory disease affecting ~2%–3% of human population [1, 2]. The major hallmarks of psoriasis are persistent inflammation and hyperproliferative epidermal keratinocytes. The incidence of psoriasis is associated with age, sex, ethnicity, and other genetic and environmental factors [3, 4]. Both the innate immune system and the adaptive immune system play an unequivocal and central role in the physiological and pathological processes of psoriasis. Innate immune cells, including myeloid dendritic cells and macrophages, release IL-12 and IL-23 to promote Th17 cells and Th1 cells to produce abundant psoriatic cytokines IL-17, IFN-*γ*, and TNF [5]. These cytokines from activated T cells induce keratinocyte hyperplasia that secrete numerous chemokines (CCL20, CXCL1, CXCL2, CXCL9, and CXCL10) to recruit more Th1 cells and Th17 cells to amplify psoriatic inflammation [6]. Therefore, some mouse models of psoriasis targeting either keratinocyte or immune cells are developed, providing strong tools to uncover the underlying molecular mechanisms of psoriasis. According to the immune mechanism underlying psoriasis, some biological agents targeting T-cell activation or cytokines, such as TNF, IL17, and IL23, are developed as therapeutics for psoriasis [4, 7]. Although lots of target therapies for psoriasis are generated, it cannot be cued and show high recurrence [8].

Salt-inducible kinases (SIKs) contain three homologous serine-threonine kinases, SIK1, SIK2, and SIK3, which belong to the AMP-activated protein kinase (AMPK) family and were first described to have a role in sensing sodium [9, 10]. Upon extracellular signals targeting membrane receptors or physiological changes, such as energy deprivation, insulin, or glucagon perturbation, SIKs can be activated to undergo phosphorylation and autophosphorylation [11]. All SIKs can be phosphorylated by LKB1; subsequent activated SIKs can phosphorylate multiple substrates, such as the transcription cofactors CRTC1-3 and HDACs, which reprogram downstream transcriptional and posttranscriptional processes [12–14]. PKA interacts with SIKs and subsequently phosphorylates SIKs to restrain their kinase activities [15]. SIKs show vital roles in multiple aspects of physiology, including metabolism, oncogenesis, and inflammation. Therefore, SIKs participate in the pathological processes of lots of human diseases, including cancer, diabetes, colitis, and sepsis [16–18]. SIK inhibitors have shown therapeutic potential for kidney injury, inflammatory diseases, and cancers [19–23]. Though SIKs emerge central roles in multiple diseases, the contribution of SIKs to the onset of psoriasis remains poorly understood.

Here, we identify that SIK1 is specifically upregulated in the imiquimod (IMQ)-induced psoriasis mouse model. Underpinning this, IL17 treatment promotes SIK1 expression in keratinocytes. Moreover, inhibition of SIK1 kinase activity with HG-9-91-01 or YKL-06-062 dramatically prevents IMQ-induced psoriasis, showing reduced inflammation and hyperproliferative keratinocytes. Mechanistically, our data demonstrate that SIK1 is required for IL17-induced activation of downstream activation of NF-*κ*B and P38 and production of proinflammatory cytokines and chemokines. Collectively, our data identify SIK1 as a critical regulator in IL17 signaling and reveal an important role for SIK1 in psoriasis pathogenesis.

## 2. Materials and Methods

### 2.1. Reagent and Antibody

Recombinant human IL-17 was purchased from novoprotein. IMQ cream was from Sichuan Mingxin. HG-9-91-01 and YKL-06-062 were purchased from MedChemExpress. Western blot was performed with the following antibodies: anti-GAPDH (sc-32233, Santa Cruz), anti-SIK1 (17370-1-AP, Proteintech), anti-*β*-actin (GB12001, Servicebio), anti-p-I*κ*b*α* (2859, Cell Signaling Technology), anti-p-P38 (4511, Cell Signaling Technology), anti-p-PJNK (4668, Cell Signaling Technology), and anti-SIK1 (phospho T182) (ab199474, Abcam).

### 2.2. Cell Culture and Generation of SIK2 Overexpressed Cell Line

293T, HaCat, and Hela cells were cultured in completed Dulbecco's modified Eagle's medium supplemented with 10% FBS, 100 U/mL penicillin, and 100 μg/mL streptomycin. To generate SIK2 overexpressed HaCat cells, PLV3-SIK2 plasmid or empty vector plasmid coupled with virus package plasmid were transferred into 293 T cells. About 60 h later, the virus was collected to infect HaCat cells. About 2 days later, puromycin was used to select positive cells.

### 2.3. Mice

Wild-type C57BL/6 mice were purchased from Gempharmatech. All mice were bred and maintained in the Institute of Translational Medicine, Shenzhen Second People's Hospital. Mice were housed under specific pathogen-free conditions. About 6–8 weeks old and gender-matched mice were used in all experiments. All animal experiments were performed according to the guidelines for the care and use of laboratory animals and were approved by the institutional biomedical research ethics committee of Shenzhen Longhua District Central Hospital.

### 2.4. IMQ-Induced Psoriasis

For the IMQ-induced psoriasis mouse model, 6–8-week-old mice were used, and a daily topical dose of 25 mg per ear IMQ was subjected to right ear for 7 consecutive days. The thickness of the ear was measured daily. For the treatment of the SIK1 inhibitors HG-9-91-01 or YKL-06-062, a daily intraperitoneal injection of 10 mg/kg of either inhibitor or DMSO control was administered to the mice. Following a 30-min interval, the mice were subsequently treated with IMQ. SIK1 inhibitors HG-9-91-01 and YKL-006-062 were dissolved in a vehicle consisting of 5% DMSO, 40% PEG300, 5% Tween 80% and 50% sterile water. DMSO control contains 5% DMSO, 40% PEG300, 5% Tween 80%, and 50% sterile water. Small molecular inhibitors or DMSO control were given 30 min before IMQ treatment.

### 2.5. Real-Time PCR

RNA was obtained from tissues or cells using Trizol according to the manufacturer's protocol. cDNA was produced by retro-transcribing RNA using a PrimeScript RT reagent kit (Takara). Real-time PCR was performed using the SYBR Premix ExTaq kit (TaKaRa) on the ABI PRISM 7500 Sequence Detection System (Applied Biosystems), according to the manufacturer's instructions. The gene expression results were normalized to the housekeeping gene *Rpl13a*. Real-time PCR primers used were provided below: human *Rpl13a* forward: CGAGGTTGGCTGGAAGTACC, reverse: CTTCTCGGCCTGTTTCCGTAG; mouse *Rpl13a* forward: GGGCAGGTTCTGGTATTGGAT, reverse: GGCTCGGAAATGGTAGGGG; mouse *Sik1* forward: TGGACGTCTGGAGCCTCGGT, reverse: AGAGTGGGGTCGGCCTGCAT; mouse *Sik2* forward: TGAGCAGGTTCTTCGACTGAT, reverse: AGATCGCATCAGTCTCACGTT; mouse *Sik3* forward: TCCCCACTTGTCACCATGAC, reverse: GAGCGATGCTGGTCAGGTAC; human *Sik1* forward: CTCCGGGTGGGTTTTTACGAC, reverse: CTGCGTTTTGGTGACTCGATG; human *Sik2* forward: AGACCACCCTCACATAATCAAAC, reverse: ATTTTCGCCTGGCTTCAGACT; human *Sik3* forward: CGTATCGGCTACTACGAGATCG, reverse: GGGGTGGCAAAGCATCTTC; human *Il6* forward: GATGAGTACAAAAGTCCTGATCCA, reverse: CTGCAGCCACTGGTTCTGT; human *Kc* forward: TCCTGCATCCCCCATAGTTA, reverse: CTTCAGGAACAGCCACCAGT; human *Ccl20* forward: GCTGCTTTGATGTCAGTGCT, reverse: TCAAAGTTGCTTGCTGCTTC; human *Cxcl2* forward: CATCGAAAAGATGCTGAAAAATG, reverse: TTCAGGAACAGCCACCAATA.

### 2.6. Western Blot

Western blot was performed as described previously [24, 25]. Briefly, cells and tissues were harvested with lysis buffer (50 mM Tris–HCl [pH 7.5], 150 mM NaCl, 1.0% Triton X-100, 1 mM EDTA, and 10% glycerol) or RIPA lysis buffer supplemented with PMSF and protease inhibitor, respectively. Cells or tissues extracts were located on ice for 30 min and centrifuged at 13,000 r.p.m for 30 min. The supernatant of extracts was transferred to a new tube and mixed with a loading buffer. Then boil the samples for 10 min. Protein samples were loaded on SDS–PAGE gels, followed by electroblotting onto PVDF membranes (Millipore). PVDF membranes were blocked with 5% milk in TBST for 1 h at room temperature and then incubated with indicated primary antibody at 4°C overnight. On second day, HRP-conjugated secondary antibodies were used to check the indicated protein expression.

### 2.7. Histology and Immunostaining

Ears from psoriasis mouse were harvested in 7 days. Tissues were fixed in 4% paraformaldehyde. Fixed ears were embedded with paraffin and sliced into 5 μm sections. Sections were stained with H&E, anti-SIK1 antibody, and Ki67. The images were obtained using with a Leica FDM2500 microscope.

### 2.8. Statistical Analysis

Statistical analysis and graph development were performed using Software Prism 8 (GraphPad Software). Two-tailed Student's *t*-test was used to compare differences between two groups. For the thickness of ears comparison, two-way ANOVA was applied. Data are shown as the mean ± standard error of the mean (SEM). Quantifications of protein expression were performed using ImageJ. All experiments were performed at least three times. Statistical significance was defined as *p* < 0.05. *⁣*^*∗*^*p* < 0.05, *⁣*^*∗∗*^*p* < 0.01, *⁣*^*∗∗∗*^*p* < 0.001.

Here, we found that SIK1 was upregulated in the IMQ-induced psoriasis mouse model. This increment may be due to a higher level of interleukin-17, which promoted the expression of SIK1 in keratinocytes. Inhibition of SIK1 kinase activity using a small molecular inhibitor (HG-9-91-01 or YKL-06-062) dramatically alleviated IMQ-induced psoriasis, showing reduced epidermal thickness, inflammation, and hyperproliferative epidermal keratinocytes. Our data demonstrated that SIK1 inhibitors HG-9-91-01 or YKL-06-062 blocked the expression of IL-17-induced proinflammatory cytokines and chemokines, including *Il6*, *Kc*, and *Ccl20*. Mechanistically, we found that SIK1 inhibitor HG-9-91-01 or YKL-06-062 suppressed the phosphorylation of I*κ*b*α* and P38. Consistently, SIK1 overexpression in keratinocytes promoted the activation of I*κ*b*α* and P38.

## 3. Results

### 3.1. SIK1 Is Upregulated in IMQ-Induced Psoriasis Model

**Figure 1:**
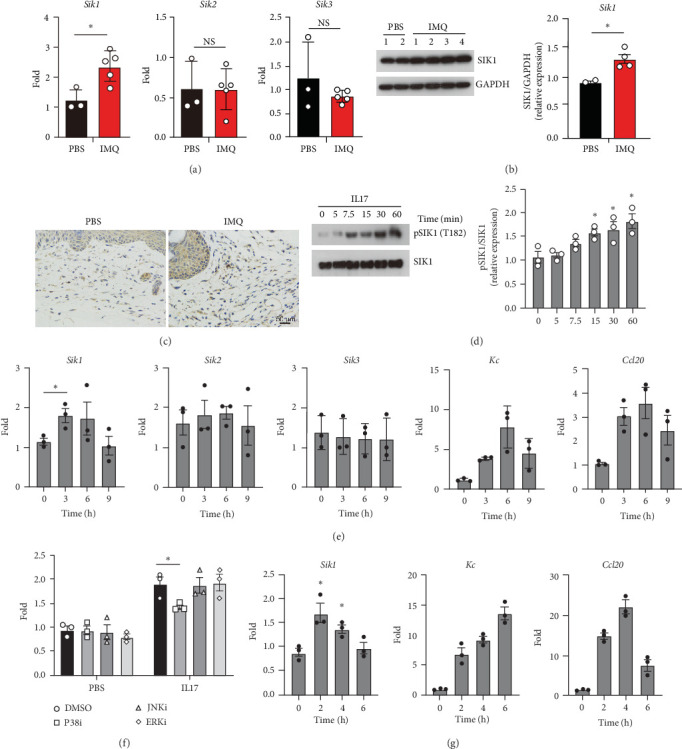
SIK1 is upregulated in IMQ-induced psoriasis. (A) Q-PCR analysis of SIKs (SIK1, SIK2, and SIK3) mRNA levels in the ears from control mice or IMQ-induced psoriasis mice. (B) Western blot and quantification of SIK1 expression in the ears from control mice or IMQ-induced psoriasis mice. Expression of SIK1 was normalized to GAPDH levels. (C) Representative images of ear sections from control mice or psoriasis mice immunostained for SIK1 antibody. The scale bar represents 50 μm. (D) Western blot and quantification of SIK1 phosphorylation in HaCat cells treated with IL-17 for the indicated time. (E) HaCat Cells were treated with recombinant IL-17 for the indicated time. Q-PCR analysis of SIKs and indicated chemokines expression. (F) HaCat cells were pretreated with MAPK inhibitor (SP600125, SB203580, and PD98059) for 30 min, then stimulated with IL-17 for 3 h. SIK1 expression were determined by qPCR. (G) HaCat Cells were treated with recombinant IL-17 for the indicated time. Q-PCR analysis of *Sik1*, *Kc*, and *Ccl20*. Results are representative of at least three independent experiments. Error bars represent SEM. Student's *t*-test, *⁣*^*∗*^*p* < 0.05, *⁣*^*∗∗*^*p* < 0.01, *⁣*^*∗∗∗*^*p* < 0.001. IMQ, imiquimod; SEM, standard error of the mean; SIK, salt-inducible kinase.

Although SIKs (SIK1, SIK2, and SIK3) have shown critical roles in multiple contexts of physiology and participate in distinct human diseases, such as cancer, diabetes, colitis, and sepsis [26], the role of SIKs in psoriasis is poorly understood. To investigate the association of SIKs and psoriasis, we employed the IMQ-induced psoriasis mouse model and evaluated the mRNA expression of SIKs. Our data showed that SIK1 was upregulated in IMQ-induced psoriasis, but SIK2 and SIK3 showed similar expression compared with control mice (Figure 1A). Western blot analysis of the SIK1 protein levels consistently showed increased expression in psoriasis (Figure 1B). In addition, we performed the immunostaining using specific anti-SIK1 antibodies and found that IMQ-induced psoriasis skin showed increased levels of SIK1 in keratinocytes compared with control (Figure 1C). Targeting IL-17/IL-17R signaling therapy has been shown to significantly alleviate psoriasis. This raises the question of whether IL-17 could activate SIK1. Our data indicated that IL-17 can induce the phosphorylation of SIK1 in keratinocytes (Figure 1D). It has been well established that keratinocytes are the target of activated T cells, including Th1, Th17, and Th22, and positively feedback to amplify psoriasis-like inflammation [3]. We hypothesize that SIK1 can be regulated by IL17 in keratinocytes. In HaCat cells, IL-17 treatment indeed induced robust expression of proinflammatory cytokines and chemokines, which is in line with previous works [27] (Figure 1E). Intriguingly, IL17 induced slightly increased expression of SIK1 in keratinocytes, which is dependent on P38 activation (Figure 1F), yet had no effect on the expression of SIK2 and SIK3 (Figure 1E). In addition to IL-17, TNF also amplifies SIK1 expression (Figure 1G). Collectively, these results indicate that IL17 promotes SIK1 expression of keratinocytes in IMQ-induced psoriasis-like mouse models.

### 3.2. Inhibition of SIK1 With HG-9-91-01 or YKL-06-062 Attenuates Skin Inflammation in IMQ-Induced Psoriasis

**Figure 2:**
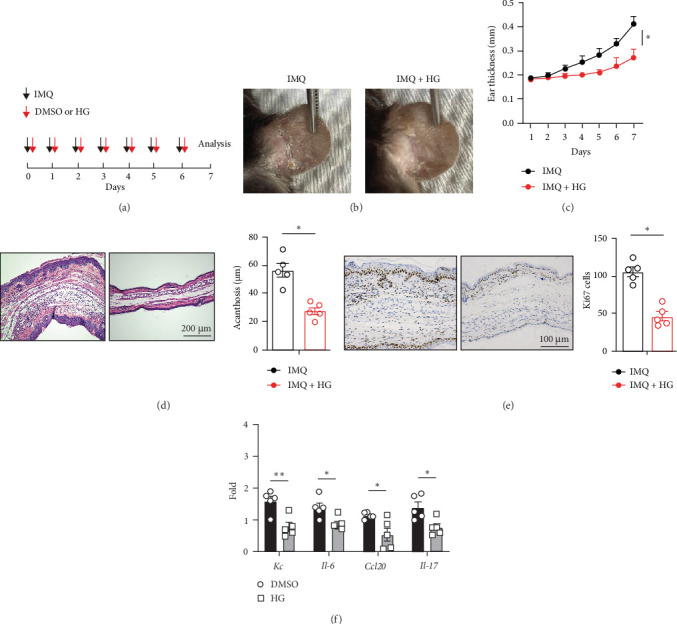
Inhibition of SIK1 with HG-9-91-01 suppresses IMQ-induced psoriasis. (A) Schematic of IMQ-induced psoriasis mouse model. About 10 mg/kg HG-9-91-01 was given intraperitoneally every day. (B) Representative images of ears from psoriasis mice or HG-9-91-01-treated psoriasis mice. (C) Ear thickness analysis of psoriasis mice or HG-9-91-01-treated psoriasis mice. (D) HE staining of ears section from indicated mice. The scale bar represents 200 μm. (E) Immunohistochemistry staining of Ki67 in ears from psoriasis mice treated with HG-9-91-01 or not. The scale bar represents 100 μm. Quantitation of Ki67-positive epidermal cells in ears from psoriasis mice was shown on the right. (F) qPCR analysis of indicated genes of ears from (A). Results are representative of at least three independent experiments. Error bars represent SEM. Student's *t*-test, *⁣*^*∗*^*p* < 0.05, *⁣*^*∗∗*^*p* < 0.01, *⁣*^*∗∗∗*^*p* < 0.001. IMQ, imiquimod; SEM, standard error of the mean; SIK, salt-inducible kinase.

Considering psoriasis mice express higher levels of SIK1, we ask whether SIK1 participates in the process of psoriasis. To evaluate the roles of SIK1 in psoriasis, we established an IMQ-induced psoriasis-like mouse model and applied SIK1 inhibitor HG-9-91-01 to inhibit SIK1 kinase activity (Figure 2A). Interestingly, the HG-9-91-01 treatment dramatically decreased the inflammatory phenotypes (erythema and scales) (Figure 2B). In line with the inflammatory phenotypes of the ear, the thickness of the ear was decreased compared with control (Figure 2C). Histological analysis of the skin lesions revealed that epidermal thickness in skin lesions was significantly reduced in mice treated with HG-9-91-01 compared with control mice (Figure 2D). Meanwhile, proliferation assessed by Ki67 staining was significantly decreased in the basal layer of the epidermis of HG-9-91-01-treated mice compared to control mice (Figure 2E). HG-9-91-01 treated mice showed mild inflammation compared to control mice treated with IMQ alone (Figure 2F).

**Figure 3:**
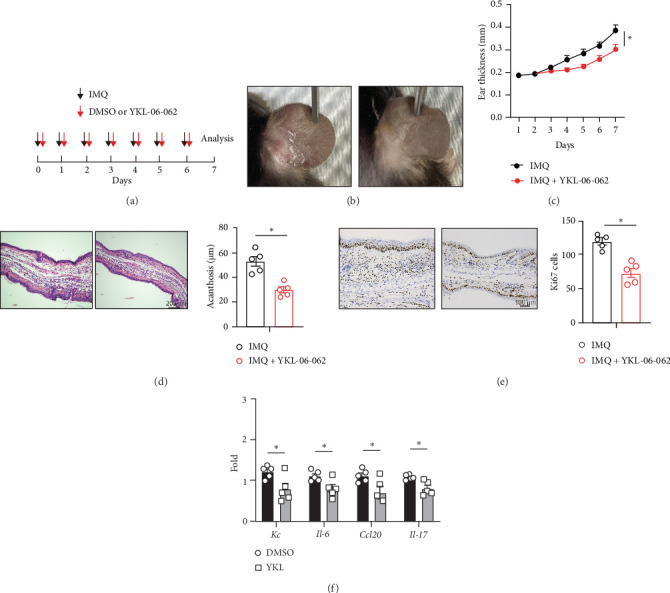
SIK1 inhibitor YKL-06-062 also alleviates IMQ-induced psoriasis. (A) Schematic of IMQ-induced psoriasis mouse model. About 10 mg/kg YKL-06-062 was given intraperitoneally every day. (B) Representative photographs of ears from psoriasis mice or YKL-06-062 treated psoriasis mice. (C) Ear thickness analysis of psoriasis mice or YKL-06-062-treated psoriasis mice. (D) HE staining of ears section from indicated mice. Scale bars, 200 μm. (E) Immunohistochemistry staining of Ki67 in ears from psoriasis mice treated with YKL-06-062 or not. Scale bars, 100 μm. Quantitation of Ki67-positive epidermal cells in ears from psoriasis mice was shown on the right. (F) qPCR analysis of indicated genes of ears from (A). Results are representative of at least three independent experiments. Error bars represent SEM. Student's *t*-test, *⁣*^*∗*^*p* < 0.05, *⁣*^*∗∗*^*p* < 0.01, *⁣*^*∗∗∗*^*p* < 0.001. IMQ, imiquimod; SEM, standard error of the mean; SIK, salt-inducible kinase.

To further confirm the effect of SIK1 on IMQ-induced psoriasis, we employed another SIK inhibitor, YKL-06-062, which also showed a high inhibitory effect on SIK1 compared with HG-9-91-01 (Figure 3A) [28]. Similarly, treatment of YKL-06-062 significantly weakened the inflammatory phenotypes (erythema and scales) (Figure 3B). The ear thickness was diminished in YKL-06-062 treated mice compared with control mice (Figure 3C). Histological examination of skin lesions indicated that epidermal thickness in skin lesions was reduced significantly in mice treated with YKL-06-062 (Figure 3D). Meanwhile, Proliferation assessed by Ki67 staining was significantly decreased in YKL-06-062 treated mice (Figure 3E). Mice treated with YKL-06-062 exhibited less cytokines and chemokines expression compared to control mice (Figure 3F). Thus, our data demonstrate that inhibition of SIK1 kinase activity alleviates skin inflammation in IMQ-induced psoriasis.

### 3.3. SIK1 Positively Regulates the Release of IL-17-Induced Proinflammatory Cytokines and Chemokines in Keratinocytes

**Figure 4:**
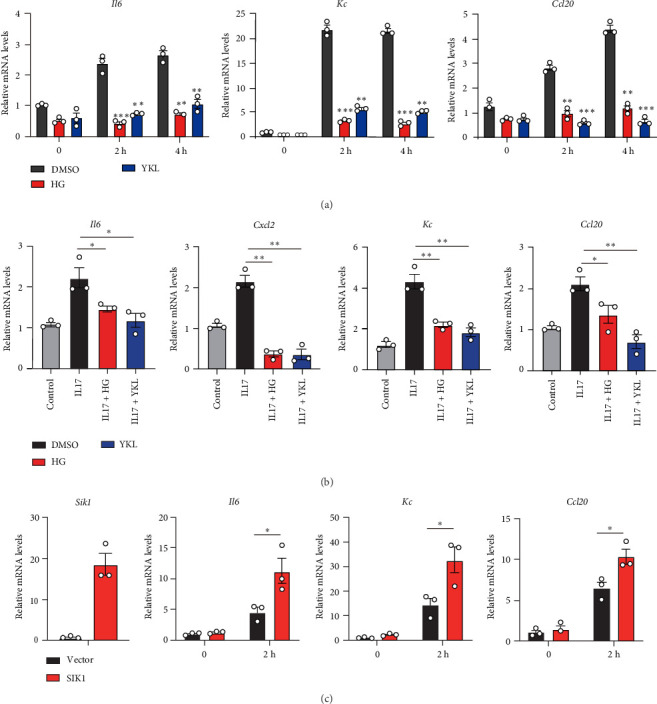
SIK1 positively regulates IL-17 signaling. (A) HaCat cells were pretreated with HG-9-91-01 or YKL-06-062 for 30 min and then stimulated with 100 ng/mL recombinant human IL-17 for the indicated time. Q-PCR analysis of expression of indicated inflammatory cytokines and chemokines. (B) Hela cells were pretreated with HG-9-91-01 or YKL-06-062 for 30 min and then stimulated with 100 ng/mL recombinant human IL-17 for the indicated time. Q-PCR analysis of expression of indicated inflammatory cytokines and chemokines. (C) HaCat cells were infected with lentivirus packaged with EV or plv3-SIK1-M2 plasmid. HaCat (EV) or HaCat (SIK1-M2) cells were treated with recombinant human IL-17 for 2 h, then Q-PCR analysis of the expression of indicated genes. Results are representative of at least three independent experiments. Error bars represent SEM. Student's *t*-test, *⁣*^*∗*^*p* < 0.05, *⁣*^*∗∗*^*p* < 0.01, *⁣*^*∗∗∗*^*p* < 0.001. SEM, standard error of the mean; SIK, salt-inducible kinase.

Though we have described the role of SIK1 in IMQ-induced psoriasis, the mechanisms underlying the function of SIK1 for psoriasis are still poorly understood. Our data showed that IL-17 can induce SIK1 expression in keratinocytes, which implies the association between SIK1 and IL-17 signaling. Therefore, we hypothesize that SIK1 may participate in IL-17 signaling and regulate its downstream production of proinflammatory cytokines and chemokines. To prove the hypothesis, we pretreated HaCat cells with HG-9-91-01 or YKL-06-062 to block SIK1 kinase activity and assessed the IL-17-induced cytokines and chemokines. Interestingly, inhibition of SIK1 kinase activity with HG-9-91-01 or YKL-06-062 blocked the production of IL-17-induced cytokines and chemokines (*Il-6*, *Kc*, and *Ccl20*) (Figure 4A). Meanwhile, HG-9-91-01 or YKL-06-062 also inhibited the IL-17-induced expression of inflammatory genes in Hela cells (Figure 4B). These data indicate that SIK1 generally regulates IL-17 signaling not only in keratinocytes but also in other epithelial cells. To further confirm the function of SIK1 on IL17 signaling, we generated SIK1 overexpression HaCat cells. Our data showed that SIK1 overexpression promoted IL17-induced expression of proinflammatory genes (*Il-6*, *Kc*, and *Ccl20*) in keratinocytes (Figure 4C). Thus, these data indicate that SIK1 positively regulates IL-17 signaling and enhances the production of IL-17 downstream proinflammatory cytokines and chemokines.

### 3.4. SIK1 Regulates IL-17 Signaling by Promoting NF-*κ*B and P38 Activation

**Figure 5:**
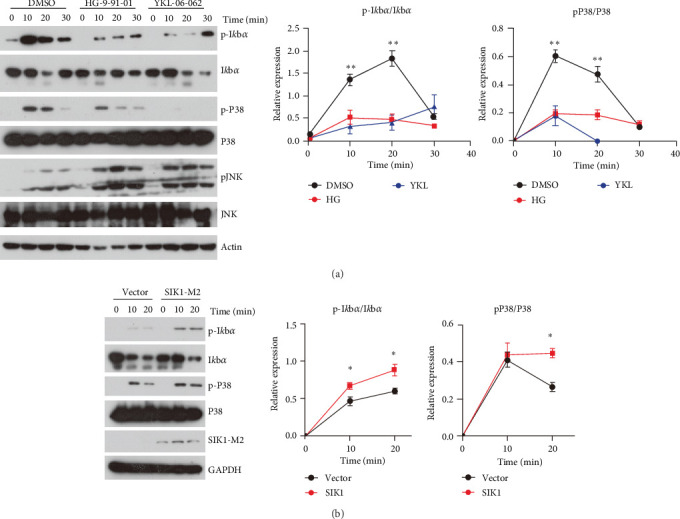
SIK1 promotes IL-17 signaling by enhancing NF-*κ*B and P38 activation. (A) HaCat cells were pretreated with HG-9-91-01 or YKL-06-062 for 30 min and then stimulated with 100 ng/mL recombinant human IL-17 for the indicated time. Western blot analysis of phosphorylation of I*κ*b*α*, P38, JNK with indicated antibody. Quantitation of p-I*κ*b*α* and p-P38 expression levels were shown on the right. (B) HaCat (EV) or HaCat (SIK1-M2) cells were treated with IL-17 for the indicated time. Western blot analysis and quantitation of phosphorylation of p-I*κ*b*α*, p-P38. Results are representative of at least three independent experiments. Error bars represent SEM. Student's *t*-test, *⁣*^*∗*^*p* < 0.05, *⁣*^*∗∗*^*p* < 0.01, *⁣*^*∗∗∗*^*p* < 0.001. SEM, standard error of the mean; SIK, salt-inducible kinase.

To further investigate how SIK1 regulates IL-17 signaling, we assessed the NF-*κ*B and MAPKs activation that is indispensable for IL-17-triggered production of inflammatory cytokines and chemokines. In HaCat cells, IL-17 induced significantly increased phosphorylation of I*κ*B*α*, whose degradation promoted NF-*κ*B activation. Pretreatment with HG-9-91-01 or YKL-06-062 dramatically blocked phosphorylation of I*κ*B*α*, which implicated that SIK1 kinase activity was required for IL-17-induced NF-*κ*B activation (Figure 5A). Moreover, we also observed decreased phosphorylation of P38 in HG-9-91-01- and YKL-06-062-treated cells, while inhibition of SIK1 showed no effect on activation of JNK (Figure 5A). These data suggest that SIK1 may positively regulate IL-17 signaling by targeting NF-*κ*B and P38 activation. Besides the utilization of the SIK1 inhibitors, we overexpressed SIK1 in HaCat cells to evaluate the function of SIK1 in IL-17 signaling. Our data showed that SIK1 overexpression increased the phosphorylation of I*κ*B*α* and P38 (Figure 5B), which was consistent with the hypothesis that SIK1 promoted NF-*κ*B and P38 activation. Taken together, our results suggest that SIK1 promotes IL-17 signaling through reinforcing the activation of NF-*κ*B and P38.

## 4. Discussion

In this study, using small molecular inhibitor HG-9-91-01 and YKL-06-062, we identify SIK1 as a critical regulator of IL17 signaling in psoriasis. We demonstrate the association between SIK1 and psoriasis, in which SIK1 expression is upregulated in keratinocytes for IMQ-induced psoriasis. Our data reveal that the upregulation of SIK1 maybe driven by proinflammatory cytokine IL-17, As IL-17 can induce the expression of SIK1 in keratinocytes. We further show that inhibition of SIK1 kinase activity with HG-9-91-01 or YKL-06-062 dramatically attenuates psoriasis-like phenotype (less immune cell infiltration, decreased proliferative epithelial cells, and less skin thickening of the ear). Mechanically, we demonstrate that SIK1 is a positive regulator of IL-17 signaling, which promotes its downstream expression of proinflammatory cytokines and chemokines. We also provide evidence supporting the role of SIK1 in IL-17 signaling as a positive regulator that functions to amplify IL-17 downstream NF-*κ*B and P38 activation. Thus, our study indicates that targeting SIK1 with small molecular inhibitors serves as a potential therapy for the treatment of psoriasis.

All SIKs are ubiquitously expressed in human tissues [10]. Compared to SIK2 and SIK3, SIK1 can be regulated by multiple stimuli, including high salt, adreno-cortico-tropichormone (ACTH) signaling [29], glucagon signaling [30], and circadian rhythms [31]. In our study, we identify that IL17 induces SIK1 expression in keratinocytes, which is responsible for more expression of SIK1 in IMQ-induced psoriasis. However, the mechanism underlying how IL17 regulates SIK1 expression is unclear. Further work needs to clarify the association between IL17 signaling and SIK1 expression. Otherwise, our results provide evidence of the SIK1 kinase activity in psoriasis that inhibition of SIK1 alleviates pathologies of psoriasis. It is unclear whether IL17 induces SIK1 kinase activation rather than induce expression. Therefore, it is further need to assess the change of SIK1 phosphorylation in IL17-stimulated keratinocytes and psoriasis tissues.

Our data reveal the therapeutic potential of SIK1 inhibitors HG-9-91-01 and YKL-06-062 for psoriasis, which significantly reduce skin inflammation of psoriasis. In addition to psoriasis, SIK1 inhibitors HG-9-91-01 and YKL-06-062 were applied to induce melanin production in human and mouse skin, potentially impacting UV protection and skin cancer risk [28]. SIK1 inhibitors target keratinocytes and trigger the transfer of melanosomes into epidermal keratinocytes. In our study, SIK1 inhibitors also target keratinocytes to participate in IL-17 signaling to inhibit cytokines and chemokines expression. In our study, SIK1 inhibitors were given intraperitoneally daily. Perhaps the psoriasis phenotypes can be more moderated by topical treatment with SIK1 inhibitors HG-9-91-01 or YKL-06-062.

SIK1 exhibits diverse functions in inflammatory signaling, which harbor proinflammatory and anti-inflammatory properties. The function of SIK1 in inflammation differs substantially in their upstream sensors and cell types. It is shown that inhibition of SIK1 in human myeloid cells induces an anti-inflammatory phenotype [32]. However, SIK1 was reported to inhibit alcohol-induced NF-*κ*B activation and apoptosis in microglia [33]. Even though SIK1 drives complex function in TLRs-induced inflammation [34], its function in IL-17 signaling is poorly understood. Our results demonstrate that SIK1 is required for IL-17-induced NF-*κ*B and P38 activation, which promote downstream proinflammatory cytokines and chemokines expression. Further work needs to be down to clarify the function of SIK1 in psoriasis with SIK1 deficiency mice. In addition, our work does not identify the targets of SIK1 in IL-17 signaling. Future study is needed to screen the substrates of SIK1, which moderate IL-17-induced NF-*κ*B and P38 activation.

Despite existing lots of treatment options, including topical therapies, phototherapy, and oral systemic therapies, psoriasis shows a high recurrence rate, and some treatments exhibit side effects interrupting patients' satisfaction. With extensive research and a deeper understanding of the pathogenesis of psoriasis in the past 20 years, biological therapies targeting TNF, IL-23/IL17, JAK1/JAK3, and PDE4 showed excellent efficacy and fewer side effects, which have been approved by FDA to treat moderate-to-severe plaque psoriasis. Secukinumab, ixekizumab, and bimekizumab are monoclonal antibodies targeting IL17A/IL17RA, which are approved in clinical use to treat psoriasis. 93% of patients given bimekizumab had PASI-75, and 79% had PASI-90 at week 12 [6]. Our work identified SIK1 as a positive regulator to promote IL-17-mediated inflammation. Targeting SIK1 kinase activity with HG-9-91-01 and YKL-061 alleviates IMQ-induced psoriasis. However, there are some limitations of therapy targeting SIK1 to treat psoriasis. SIK1 also participates in other processes of human diseases, especially in diabetes and tumorigenesis. Therefore, inhibition of SIK1 kinase activity may promote tumorigenesis or disrupt gluconeogenesis and lipid metabolism [29, 35, 36]. Otherwise, SIK1 inhibitors were shown to inhibit TLR-induced inflammation, which may lead to adverse events such as infection [37, 38]. Collectively, further work needs to clarify the underlying mechanisms by which SIK1 regulates IL17 signaling and the role of SIK1 in the pathogenesis of psoriasis.

## 5. Conclusion

Here, we found that SIK1 was upregulated in the IMQ-induced psoriasis mouse model. IL-17 could promote the expression of SIK1 in keratinocytes in vitro. Inhibition of SIK1 kinase activity using a small molecular inhibitor (HG-9-91-01 or YKL-06-062) dramatically alleviated IMQ-induced psoriasis. Our data demonstrated that SIK1 inhibitors HG-9-91-01 or YKL-06-062 blocked the expression of IL-17-induced proinflammatory cytokines and chemokines. Mechanistically, we found that SIK1 inhibitor HG-9-91-01 or YKL-06-062 suppressed the downstream phosphorylation of I*κ*b*α* and P38 of IL-17 signaling. Our results reveal that SIK1 participates to promote IL17-induced signaling through enhancing activation of NF-*κ*B and MAPKs and exacerbates psoriasis-like skin inflammation. Thus, inhibition of SIK1 presents a potential new therapeutic target for psoriasis.

## Data Availability

The datasets generated during the current study are available from the corresponding author upon reasonable request.
